# Why is Korean girls’ suicidal ideation rate higher than boys’ rate? The role of gender heterogeneity in peer groups

**DOI:** 10.1371/journal.pone.0290072

**Published:** 2023-09-06

**Authors:** Youngjoon Bae, Jaein Lee

**Affiliations:** 1 Population Research Center, The University of Texas at Austin, Austin, Texas, United States of America; 2 Department of Sociology and Criminology, Arkansas State University, Jonesboro, Arkansas, United States of America; Sunway University, MALAYSIA

## Abstract

Girls typically show much lower suicide rates than boys in most OECD countries. However, in South Korea, the suicide rate of girls almost reaches that of boys. Moreover, Korean girls’ suicide rate is remarkable even among other advanced countries. One potential approach to explaining Korean girls’ relatively high suicide rate is to investigate how their peer groups affect suicidal ideation, but this approach has rarely been explored in Korean adolescents. We tested how the gender heterogeneity of peer groups is associated with suicidal ideation by analyzing 2,990 adolescents from the 2018 Korean Children and Youth Well-Being Index Survey. For the analysis, logistic regression models with survey weights were used. The analysis revealed that adolescents with different-gender friends were associated with a higher likelihood of suicidal ideation than those with exclusively same-gender friends when adjusting for covariates. In addition, an analysis stratified by gender found that this association was significant only among girls. Furthermore, the protective power of having a mentor against suicidal ideation was significantly lower in girls with male and female friends than in girls with only female friends. The findings suggest a less protective role of different-gender peer groups for suicidal ideation among girls. During adolescent suicidality consultations, school counselors and practitioners should pay attention to the characteristics of adolescents’ peers, particularly their gender.

## Introduction

In South Korea (hereafter, Korea), suicide is a leading cause of adolescent death [1]. Although boys tend to report higher suicide rates than girls in most countries of the Organization for Economic Co-operation and Development (OECD), the rates are relatively similar between boys and girls in Korea [2, 3]. Additionally, the gap between boys and girls became narrower after 2006 [2]. Korean girls’ suicide rate is remarkable compared with other advanced countries [2]. What are the reasons for girls’ exceptionally high suicide rate, especially compared to Korean boys?

This question is not easy to answer because data on suicide are not typically linked to sociodemographic information about those who die by suicide. Therefore, the present paper, as a pilot study, examines the potential reasons for higher *suicidal ideation* in Korean girls because suicidal ideation should precede suicide. Indeed, a recent report by the Statistics Research Institute in Korea indicated that girls showed higher rates of suicidal ideation (16.1% vs. 9.5%) and suicidal attempts (2.9% vs. 1.5%) than boys in 2021 [4]. The suicide rates in 2021 were comparable between boys (7.3 deaths per 100,000) and girls (6.9 deaths per 100,000) [5]. The current study also seeks to elucidate a *group mechanism* of higher suicidal ideation among Korean girls. The collective reason would more properly explain Korean girls’ suicidal ideation and, potentially, suicide.

This study provides a unique contribution to the field of adolescent suicidal ideation by focusing on a collective mechanism of Korean girls’ high suicidality, which appears distinctive among advanced countries. Prior studies shed light on the phenomenon, but their explanations tended to not be specific to girls. We suggest that girls with male and female friends have a greater likelihood of suicidal ideation than girls with only female friends. As a potential mechanism, we note weaker social support among girls with male and female friends. The present paper also extends understanding of how the gender composition of peer groups affects suicidal ideation. This is important, particularly in Korea, because the country may experience a change in the gender composition of peer groups due to the expansion of coeducational schools. Most studies have investigated romantic relationships and their consequences among adolescents. To our knowledge, this is the first study to examine the association between the gender composition of peer groups and suicidal ideation among Korean adolescents.

### Risk factors for suicidal ideation

Most prior studies in the field have examined the risk factors for *suicidal ideation* rather than *suicide*. Some studies have used the terms suicidality or suicidal thoughts instead of suicidal ideation. Because suicidal ideation is broadly defined as thoughts of engaging in suicide [6, 7], the present paper uses these terms interchangeably. Findings from research on suicidal ideation suggest that one of its robust predictors is depression [8, 9], but it is significant in both male and female adolescents [9–12]. Therefore, it cannot be a distinctive risk factor for suicidality among girls. Recent studies have also found that experiences of bullying, sleep quality, the consumption of energy drinks, and the timing of sexual initiation were associated with suicidal ideation, but these are also risk factors for both boys and girls [13–17]. In contrast, smoking experience [10], conflicts with parents [18], psychological problems of parents [19], and early menarche [20] were positively associated with suicidality among girls. However, these reasons may not be sufficient to account for the high rates of suicidality in Korean girls. Smoking is far more prevalent among boys, and conflicts with parents would show wide variation among girls. Additionally, it is unreasonable to hypothesize that girls’ parents have a greater likelihood of psychological problems than boys’ parents do. In other words, these factors, except for menarche, are not specific to girls. To be sure, menarche belongs to girls. Nevertheless, there is no reason that a greater proportion of Korean girls experience early menarche compared to girls from other developed countries, although a growing number of studies suggest potential factors, such as soft drink consumption [21], milk intake [22], household income [23], and childhood abuse [24], that expedite the first occurrence of menstruation.

### The peer group of adolescents and its stronger supportive role among girls with female friends

The present paper examines the peer groups of adolescents because adolescents tend to have same-gender friends, and their behaviors are greatly influenced by their friends. The higher rate of suicidality among Korean girls may be explained by this group mechanism. Adolescents’ weak social support from friends tends to have an accelerated effect on the association between depression and suicidal ideation [25]. Several studies using Korean adolescent samples have found consistent results showing the protective role of social support. If boys and girls have conflicts with friends, they are likely to have suicidal thoughts [18]. Furthermore, weak support from a few friends [26, 27] or substantial alienation from classmates in school life [11] are positively associated with suicidality.

This supportive role tends to be significant among girls [28–30]. Girls are more emotionally responsive to their friends [31, 32], and they are more likely than boys to sacrifice their own needs to privilege the demands of others [33, 34]. Although girls prioritize their friends’ needs, they do not lack mutual support from their female friends. Girls lacking this supportive relationship due to social isolation have a greater risk of detrimental mental health outcomes, including suicidality [35, 36].

In this context, social support may likely be magnified in the members of girls’ cliques. The role of friends of the same gender is more prominent among girls [37–39]. All-girl groups had the closest relationship, while mixed-gender groups had weakly tied friendships. Mixed-gender groups are also associated with a greater likelihood of deviant behavior [40]. Furthermore, a supportive attitude may be interpreted differently among boys. One study reported that some boys tried not to be emotional or supportive to avoid being ridiculed, criticized, and rejected by their male friends [41]. Boys also used humor rather than supportive approaches during problem talk; this was accepted as closeness only for boys [32]. A feminine role, such as emotional expressiveness, makes it easy for girls to share social support. In contrast, a masculine role, such as emotional control, makes it difficult for boys to seek and obtain social support [42].

In summary, girls whose friends consist of only female friends (gender homogeneity of peer groups) are likely to receive greater social support, and this support may play a protective role against suicidal ideation. Therefore, the current study argues that girls with male and female friends (gender heterogeneity of peer groups) may experience relatively weakened social support compared to their counterparts with only female friends. Furthermore, social support may effectively decrease the likelihood of suicidality among girls with only female friends. Social support among girls with male and female friends is likely to be weakened by their male friends, who may respond to emotional communication with less serious attitudes. In this circumstance, social support would not be actively stimulated.

### The Korean context

The meaning of the gender heterogeneity of peer groups is a critical point of the current study, and it should be discussed in a Korean context. This is because having different-gender friends has specific implications in Korea. For instance, an old Korean idiom says that a boy and a girl should not sit together after they turn 7. This example emphasizes the significance of gender segregation even during early childhood. This culture has changed rapidly, and now many Koreans acknowledge the value of gender-integrated education, at least in elementary schools. However, under the expansion of the coeducational system in middle and high school, a growing number of students and parents doubt the value of the coeducational system due to lower test scores for college entrance in coeducational high schools, although findings in the field have reported mixed results [43, 44].

Homophily, which means the tendency to have relationships with similar others, is a strong mechanism for constructing peer groups at the individual level, especially among adolescents in Korea [45]. Therefore, having a different-gender friend may be easily noticeable by others and tends to be regarded as having a romantic relationship. While the gender heterogeneity of peer groups does not necessarily indicate a romantic relationship, several studies have reported that such relationships occur more frequently in mixed-gender schools [46] and are associated with adverse outcomes such as lower ego identity and ego resilience [47].

### Hypotheses

As mentioned earlier, data on adolescent suicide are extremely restricted. Additionally, information on the friend networks of suicide attempters or suicide completers is difficult to collect; even the data collection can be considered unethical. This is because suicide attempters and their friends may undergo traumatic experiences. Several studies have reported the contagion effect of suicide among adolescents [35, 48, 49]. This has also been reported among Korean adolescents [50]. Recollection of a stressful event may facilitate emotional distress, suicidal ideation, and even suicide attempts. This is one reason for the lack of data. However, a review article suggests counterevidence that encourages asking about suicidality [51]. Data on this information will strengthen understanding of the causal mechanisms of how a friend’s suicidal behaviors affect his or her peer groups in terms of suicidal ideation and suicide attempts.

Currently, as an exploratory step, the present study investigated the association between the gender heterogeneity of peer groups and *suicidal ideation*. Based on the literature, we constructed the following hypotheses.

*H1*: *Girls with close female and male friends (gender heterogeneity of peer groups) have a greater likelihood of suicidal ideation than girls with only close female friends (gender homogeneity of peer groups)*.
*H2*: *The probability of suicidal ideation being reduced by having a mentor is lower among girls who have only close female friends (gender homogeneity of peer groups) than girls with close female and male friends (gender heterogeneity of peer groups)*.

## Materials and methods

### Data

This study uses data from the 2018 Korean Children and Youth Well-Being Index Survey, which includes nationally representative data and has surveyed different participants every year since 2009. Part of the survey collected data from middle and high school students between March 7th, 2018, and April 6th, 2018. Participants were chosen by probability proportional to size (PPS) sampling with regard to school, region, and sex while considering a population unit as a class. The survey administrators deidentified the data; thus, users cannot link participants with their actual identities. These publicly available data can be downloaded from the following website: https://kossda.snu.ac.kr/handle/20.500.12236/23362. This research complies with the ethical standards of STrengthening the Reporting of OBservational studies in Epidemiology (STROBE), and the authors have provided a completed STROBE checklist as S1 Checklist.

This article tested the most recent available year at the time of this research. Currently, the survey provides data for 2021, but we did not use this data because it was collected during the pandemic. During the pandemic, students attended classes remotely in Korea, and their interaction with friends was likely to be different from regular face-to-face contact. Thus, the relationship between peer characteristics and suicidal ideation may have differed from the pre-pandemic period. We assume that this difference may be an ad hoc case, although it would provide unprecedented insight into how weakened in-person social contact is associated with suicidal ideation. However, this is an area for future research. The present study aimed to examine the group mechanism of Korean girls’ high suicidal ideation, which has been observed at least since 2013 [52].

In Korea, several datasets examine suicidal behaviors among adolescents. However, this dataset is the only public source that simultaneously asks about suicidal behaviors and friendship networks from a nationally representative sample. One of the strengths of this dataset is that it reports not only the network of friends but also the sociodemographic traits of friends, such as their gender, social class, and academic achievement. Approximately 20% of respondents did not provide their best friends’ academic grade. However, a sensitivity test excluding this item repeatedly revealed similar results in full models adjusting for covariates. After removing items with missing values, the analytic sample included 2,990 students in middle and high schools, consisting of 1,602 boys and 1,388 girls.

### Measures

#### Suicidal ideation

The outcome variable was whether a person had an impulse to commit suicide. The survey asked whether participants had ever thought about their suicide by impulse (*yes* = 1; *no* = 0), and a follow-up question asked for their reasons. Among the reasons for suicidality, ’conflicts with parents’ was most prevalent for both boys (39.5%) and girls (38.9%). ‘Another reason’ followed for boys (17.8%) and girls (19.6%). ‘Decline in school grades’ appeared important for boys (14.2%) and girls (12.4%). ‘Conflicts with friends’ also emerged as significant for both boys and girls, but the percentages indicated a remarkable gap (6.7% for boys and 12.9% for girls). The reference group was adolescents who had never had an impulse to commit suicide.

#### Gender, gender heterogeneity of peer groups, and mentor availability

The key independent variables were the gender of the respondent (employed as girls), the gender heterogeneity of the peer groups, and the availability of mentors in the first analysis. Gender was omitted in the second analysis stratified by gender. For girls, a value of 1 was assigned, while boys were coded as the reference group. Respondents provided the names of their best friends (up to 7) along with their friends’ gender (male or female), social class (upper, middle, or lower), and academic achievement (upper, middle, or lower). The gender heterogeneity of peer groups was calculated as 1 if any of the respondent’s friends had a different gender than the respondent. If a respondent had only same-gender friends, a value of 0 was given. Heterogeneity in social class and academic achievement was calculated similarly for comparison. As a proxy measure of social support, whether a respondent had a mentor was included. If a respondent had a mentor, a value of 1 was given; otherwise, 0 was assigned.

#### Covariates

Covariates thought to influence adolescents’ suicidal ideation were considered from 7 perspectives: relationships with friends and parents, experiences of bullying, delinquent behavior, stress, social activities, health behavior and conditions, and demographic traits. Regarding friend relationships, four indicators of relationships with best friends were included: *I feel ashamed to talk about my problems with them*, *I feel lonely even while spending time with them*, *I feel anger due to them*, and *They do not care about my sorrow*. Each item was measured on a 5-point Likert scale. Higher scores indicated a worse relationship. In addition, the number of best friends was included. The relationship with parents (father and mother separately) and the relationship between parents were included on a 5-point Likert scale. Higher scores indicated a better relationship. Additionally, respondents were asked whether they felt loneliness and responded on a 5-point scale. Higher scores demonstrated more loneliness.

Experiences of bullying were included. The survey separately measured whether a respondent experienced being bullied and whether the respondent participated in bullying behavior. If the respondent answered yes, a value of 1 was assigned to each item; otherwise, a value of 0 was assigned. Delinquent behavior was also considered, such as drinking, smoking, and sexual intercourse. The respondents answered each item as yes or no. If they had the experience, 1 was coded; otherwise, a value of 0 was given. Two sets of stress variables were considered. First, three variables of stress resulting from relationships with parents were included: *stress due to conflicts with parents*, *stress due to parental interference*, and *stress due to lack of understanding from parents*. Second, two variables related to schoolwork were employed: *stress from bad grades* and *stress from assignments and exams*. These stress variables were measured on a 5-point scale. Higher scores indicated a higher level of stress. Multiple types of social activities were considered, such as participation in club activities, religious attendance, volunteer activities, civic activities, celebrity fan clubs, and games. All variables were measured on a 5-point scale, and higher scores indicated more frequent participation.

Health behavior and conditions were considered through workouts, sleep time, self-reported health, pharmacy visits, and hospitalization. The frequency of workouts per week was presented in a range from 0 to 7. Average hours of sleep were used and centered on a group mean. Because sleep time differs between middle schoolers and high schoolers, the separate group mean was applied. Self-reported health was included on a 5-point scale; higher scores showed better health. Pharmacy visits within 2 weeks and hospitalization within a year were taken into consideration. If the respondent had this experience, 1 was coded; otherwise, a value of 0 was assigned. Sociodemographic variables such as school grade, academic achievement, and social class were taken into account. If the respondent was a middle school student, 1 was assigned, while high school students were coded as a reference group. Academic achievement and social class were included separately on a three-point Likert scale. Higher scores showed better achievement and higher social class.

### Statistical analyses

A logistic regression framework with survey weights was adopted for the binary dependent variable of whether a person had an impulse to commit suicide. Model 1 tested the associations between three measures of peer heterogeneity (gender, social class, and academic achievement) and the odds of suicidality while considering the respondent’s gender. Model 2 added mentor availability. Model 3 adjusted for all covariates. Additionally, these models were applied to subsamples stratified by gender. Interaction terms between the respondents’ gender and the gender heterogeneity of friends were initially considered. However, some scholars have argued that estimates using interaction terms in nonlinear models could be problematic [53]. For interaction terms in logistic regression models, caution is required when interpreting the size and direction of the estimates and statistical significance. Instead, a delta method for statistical significance can be used [54, 55]. To do so, the "Margins" command in Stata was utilized to visualize the simultaneous associations of respondents’ gender (or mentor availability) and the gender heterogeneity of peer groups with suicidality. Diagnostic tests were employed and reported as supporting information. No specification errors or multicollinearity were found. All analyses were estimated using Stata 17.

## Results

### Demographic characteristics

Table 1 reports descriptive statistics for the variables used in the analysis. The estimates were separated by gender to determine whether the two groups had significantly distinct differences.

**Table 1:** Descriptive statistics for boys and girls (with survey weights).

	Boys		Girls		*P value*
Number of respondents	1,602		1,388		
	Mean/%	S.D.	Mean/%	S.D.	
Suicidal ideation (%)	18.5		37.7		***
Number of best friends (0–7)	5.3	2.2	5.1	2.0	**
Gender heterogeneity of peer groups (%)	21.9		23.1		
Academic achievement heterogeneity of peer groups (%)	78.7		77.9		
Social class heterogeneity of peer groups (%)	53.5		45.6		***
I have someone to discuss my concerns (%).	95.0		94.8		
*Relationship with best friends (all*: *1–5 scale)*					
I feel ashamed to talk about my problems with them.	1.9	1.0	2.1	1.0	**
I feel lonely even while spending time with them.	1.5	0.8	1.7	1.0	***
I feel anger due to them.	2.3	1.1	2.5	1.1	***
They do not care about my sorrow.	2.0	1.0	2.0	1.0	
Good relationship with father (1–5)	4.3	0.9	4.3	1.0	
Good relationship with mother (1–5)	4.6	0.7	4.6	0.7	
Good relationship between father and mother (1–5)	4.2	0.9	4.2	0.9	
Loneliness (1–5)	2.1	1.2	2.4	1.2	***
Bullied (%)	5.0		6.1		
Bullying (%)	10.4		3.1		***
Drinking ever (%)	25.4		17.2		***
Smoking within a week (%)	6.6		1.1		***
Sexual intercourse ever (%)	3.2		1.3		***
Stress from conflicts with parents (1–5)	2.6	1.3	2.9	1.3	***
Stress from parental interference (1–5)	2.3	1.2	2.4	1.2	
Stress from lack of understanding by parents (1–5)	2.2	1.2	2.5	1.3	***
Stress from bad grades (1–5)	2.6	1.3	3.0	1.3	***
Stress from assignments or exams in school (1–5)	2.9	1.3	3.3	1.3	***
Club activities (1–5)	3.3	1.4	3.4	1.4	*
Participation in religious services (1–5)	2.1	1.4	2.0	1.4	
Volunteering activities (1–5)	2.7	1.3	2.9	1.3	***
Participation in civic activities (1–5)	1.7	1.0	1.7	1.0	
Celebs fan club (1–5)	1.7	1.1	2.6	1.5	***
Gaming (1–5)	3.1	1.3	2.2	1.3	***
Workout days a week (0–7)	3.2	2.0	2.7	1.7	***
Sleep time (Centered on group means: middle and high school, each)	0.1	1.2	-0.2	1.2	***
Self-reported health (1–5)	4.2	1.0	4.0	1.0	***
Visit to a pharmacy within two weeks (%)	26.9		32.6		***
Hospitalization within a year (%)	10.0		5.4		***
Middle schoolers: ref. high schoolers (%)	42.0		42.8		
Academic achievement (1–3)	2.05		2.04		
Social class (1–3)	1.86	0.5	1.94	0.4	***

Note. For categorical variables, percentages are presented. For continuous variables, means and SDs are presented.

*** p<0.001

** p<0.01

* p<0.05

In general, boys and girls showed significant differences in most variables. As expected, a much higher percentage of girls (37.7%) than boys (18.5%) had an impulse to commit suicide. The size of the best friend group for girls (5.1) was marginally but significantly smaller than that for boys (5.3). Gender was the most potent boundary cluster of friendship. Approximately 20% of male and female adolescents had friends of different genders. In other words, approximately 80% of boys and girls had all-male peer groups and all-female peer groups, respectively. Adolescents tended to have friends regardless of their academic achievement. Approximately 80% had friends with different grades from the respondents. Boys and girls did not indicate significant differences in either the gender or academic grade composition of their friends. However, a smaller proportion of peer groups among female adolescents comprised those with a different social class (45.6%) compared to their male counterparts (53.5%).

Girls revealed more negative emotions toward their best friends than boys did. For example, the mean score of whether they felt ashamed to talk about their problems with their friends was 2.1 among female adolescents and 1.9 among male adolescents. Similarly, girls reported higher mean scores than boys for the following statements: I feel lonely while being together with my best friends (1.7 for girls vs. 1.5 for boys) and I feel anger due to them (2.5 for girls vs. 2.3 for boys). However, a preliminary analysis indicated that these variables were not associated with suicidal ideation. Most girls and boys had someone to discuss their concerns with (94.8% and 95%, respectively).

Girls perceived greater stress from their relationship with their parents. Female adolescents indicated higher levels of stress from parental conflicts (2.9 for girls vs. 2.6 for boys) and a lack of understanding from parents than male adolescents did (2.5 for girls vs. 2.2 for boys). However, girls were less likely to be involved in delinquent behaviors than boys were. These included bullying (3.1% for girls vs. 10.4% for boys), drinking ever (17.2% for girls vs. 25.4% for boys), smoking within a week (1.1% for girls vs. 6.6% for boys), and sexual intercourse ever (1.3% for girls vs. 3.2% for boys). There were no significant differences in the experience of being bullied between the groups. Girls indicated worse conditions in other potential risk factors for suicidal ideation than boys did. These included loneliness (2.4% for girls vs. 2.1% for boys), stress from bad grades (3.0% for girls vs. 2.6% for boys), stress from assignments and exams (3.3% for girls vs. 2.9% for boys), and mean sleep time (-0.2 for girls vs. 0.1 for boys).

### The association between gender heterogeneity of peer groups and suicidal ideation among adolescents

Table 2 reports the odds ratios of whether adolescents had an impulse to commit suicide. AIC and BIC decreased when adding variables, which shows that the model fit is the best in the full model. Girls had a higher likelihood of suicidal ideation than boys in all models, and the relationship was statistically significant at p < 0.001. Specifically, girls had approximately 2.6–2.7 times the odds of suicidality of boys. Gender heterogeneity of peer groups showed statistical significance only among the measures of peer group heterogeneity. Adolescents with different-gender friends tended to have a higher probability of suicidal ideation than those with friends of the same gender only (OR = 1.35, p < 0.01). This relationship remained after adjustment for mentor availability (OR = 1.37, p < 0.01) and covariates (OR = 1.37, p < 0.05). Having a mentor and suicidal ideation revealed a significant negative association (OR = 0.30, p < 0.001). However, considering covariates, the odds ratio and statistical significance were attenuated (OR = 0.57, p < 0.01).

**Table 2:** Logistic regression estimating odds ratios of suicidal ideation among boys and girls (with survey weights).

	Suicidal ideation, *Odds ratios (95% CIs)*		
	Boys and Girls (N = 2,990)		
	Heterogeneity Model	Mentor Model	Full Model
Girls (ref. boys)	2.70***	2.70***	2.62***
	(2.26–3.23)	(2.25–3.23)	(2.07–3.32)
*Heterogeneity of peer groups*			
I have a friend of a different gender.	1.35**	1.37**	1.37*
(ref. all the same gender)	(1.10–1.66)	(1.11–1.68)	(1.08–1.74)
I have a friend from a different academic grade group.	0.99	1.02	0.95
(ref. all the same group)	(0.80–1.23)	(0.82–1.26)	(0.75–1.21)
I have a friend of a different social class.	1.18	1.20	1.08
(ref. all the same social class)	(0.99–1.41)	(1.00–1.43)	(0.88–1.33)
I have someone to discuss my concerns.		0.30***	0.57**
(ref. no mentor)		(0.21–0.42)	(0.38–0.85)
AIC	1.103	1.087	0.914
BIC	-20602	-20658	-20949

Note. The full model adjusts for the number of best friends, relationship measures with the best friends, experience of bullying or bullying behavior, relationship with parents, loneliness, drinking, smoking, sexual intercourse, stress from academic grades or exams, gaming, workouts, social activities, social class, middle school or high school, academic achievement, self-reported health, pharmacy visits, hospitalization, and sleep time.

For comparison of models, AIC and BIC are reported.

*** p < 0.001

** p < 0.01

* p < 0.05

**Fig 1:**
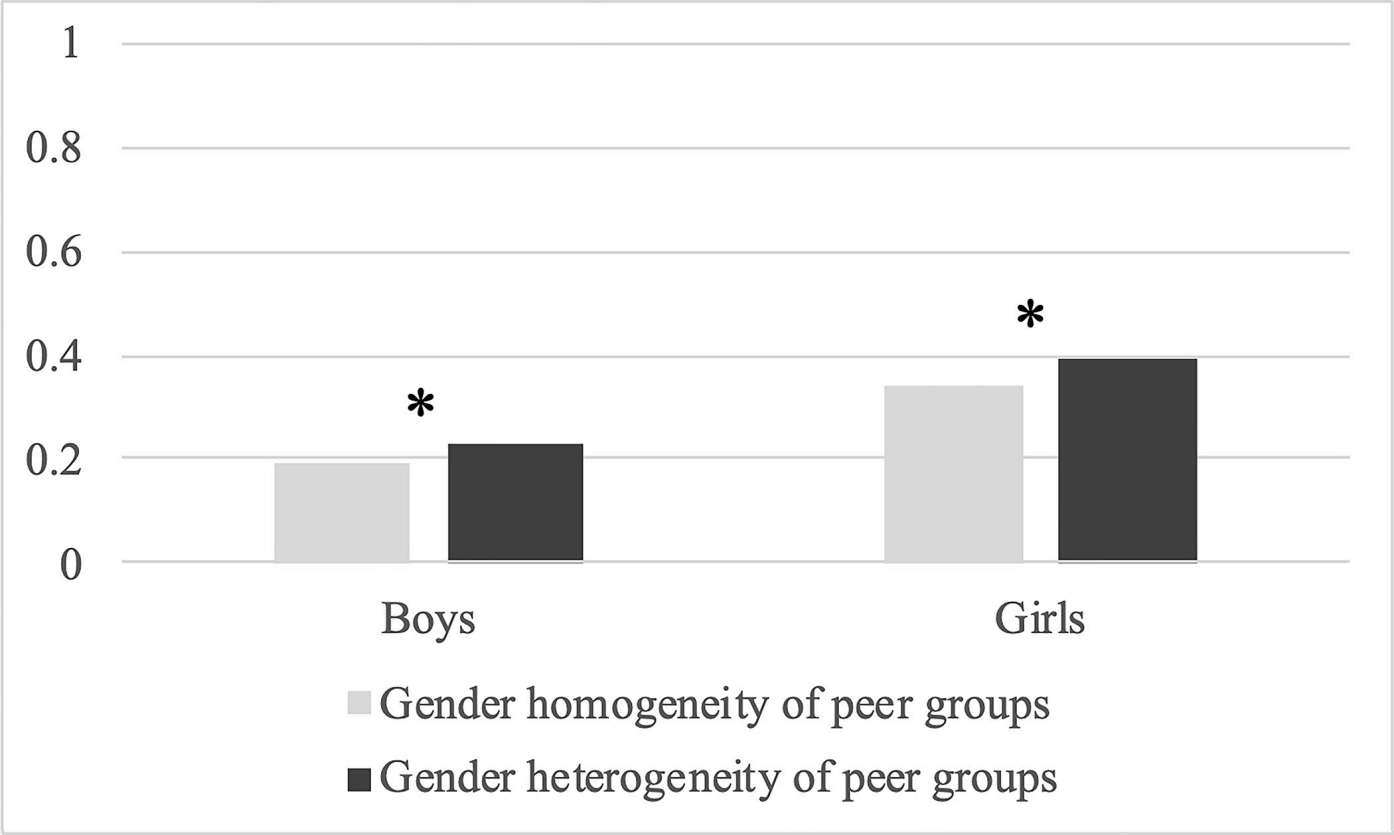
Predictive margins of suicidal ideation by gender and gender heterogeneity of peer groups. * p < 0.05.

Fig 1 visualizes how a respondent’s gender and the gender heterogeneity of peer groups were associated with the likelihood of suicidality. Regardless of the gender composition of the peer groups, girls had a higher likelihood of suicidal ideation than boys. Among girls, those with male and female friends indicated a higher likelihood of suicidality than those with female friends only. This pattern was also found among boys. Boys with male and female friends showed a greater likelihood of suicidal ideation than those with only male friends. These results suggest that the current analysis finds potential evidence for the role of different-gender friends and an increased likelihood of suicidal ideation among both girls and boys.

### The association between gender heterogeneity of peer groups and suicidal ideation among girls and the differential role of a mentor

Table 3 reports how the gender heterogeneity of the peer groups is associated with suicidal ideation for boys and girls. The AIC and BIC scores show that the full model in each subsample is the best fit. Among boys, the heterogeneity of peer groups with regard to gender, social class, or academic achievement did not reveal significant associations with suicidal ideation. Boys with a mentor tended to have a lower likelihood of suicidal ideation than those without a mentor (OR = 0.42, p < 0.01). However, this association disappeared after adjustment for covariates. In contrast, among girls, gender heterogeneity of peer groups was associated with an increased likelihood of suicidal ideation (OR = 0.68, p < 0.01). Furthermore, the significance was maintained when adjusting for mentor availability (OR = 0.67, p < 0.01) and other covariates (OR = 0.64, p < 0.01). In other words, girls with male and female friends tended to have a higher likelihood of suicidal ideation than girls with female friends only. Additionally, girls with a mentor showed a lower likelihood of suicidal ideation in the full model (OR = 0.44, p < 0.01). These results for girls suggest evidence supporting *Hypothesis 1*.

**Table 3:** Logistic regression estimating odds ratios of suicidal ideation among boys and girls separately (with survey weights).

	Suicidal ideation, *Odds ratios (95% CIs)*					
	Boys (N = 1,602)			Girls (N = 1,388)		
	Homogeneity Model	Mentor Model	Full Model	Homogeneity Model	Mentor Model	Full Model
*Heterogeneity of peer groups*						
I have a friend of a different gender.	1.21	1.15	1.23	1.48**	1.48**	1.55**
(ref. all the same gender)	(0.88–1.65)	(0.89–1.68)	(0.86–1.77)	(1.12–1.95)	(1.12–1.97)	(1.12–2.16)
I have a friend from different academic grade groups.	1.10	1.12	1.08	0.92	0.95	0.88
(ref. all the same group)	(0.79–1.54)	(0.80–1.56)	(0.74–1.58)	(0.70–1.22)	(0.71–1.27)	(0.63–1.23)
I have a friend of different social classes.	1.15	1.16	1.06	1.19	1.22	1.10
(ref. all the same social class)	(0.87–1.51)	(0.88–1.52)	(0.77–1.45)	(0.94–1.51)	(0.96–1.55)	(0.83–1.45)
I have someone to discuss my concerns.		0.42**	0.76		0.22***	0.44**
(ref. no mentor)		(0.25–0.72)	(0.41–1.41)		(0.13–0.36)	(0.25–0.79)
AIC	0.941	0.937	0.789	1.293	1.263	1.086
BIC	-10292	-10301	-10342	-8228	-8,271	-8326

Note. The full model adjusts for the number of best friends, relationship measures with the best friends, experience of bullying or bullying behavior, relationship with parents, loneliness, drinking, smoking, sexual intercourse, stress from academic grades or exams, gaming, workouts, social activities, social class, middle school or high school, academic achievement, self-reported health, pharmacy visits, hospitalization, and sleep time.

For comparison of models, AIC and BIC are reported.

*** p < 0.001

** p < 0.01

* p < 0.05

**Fig 2:**
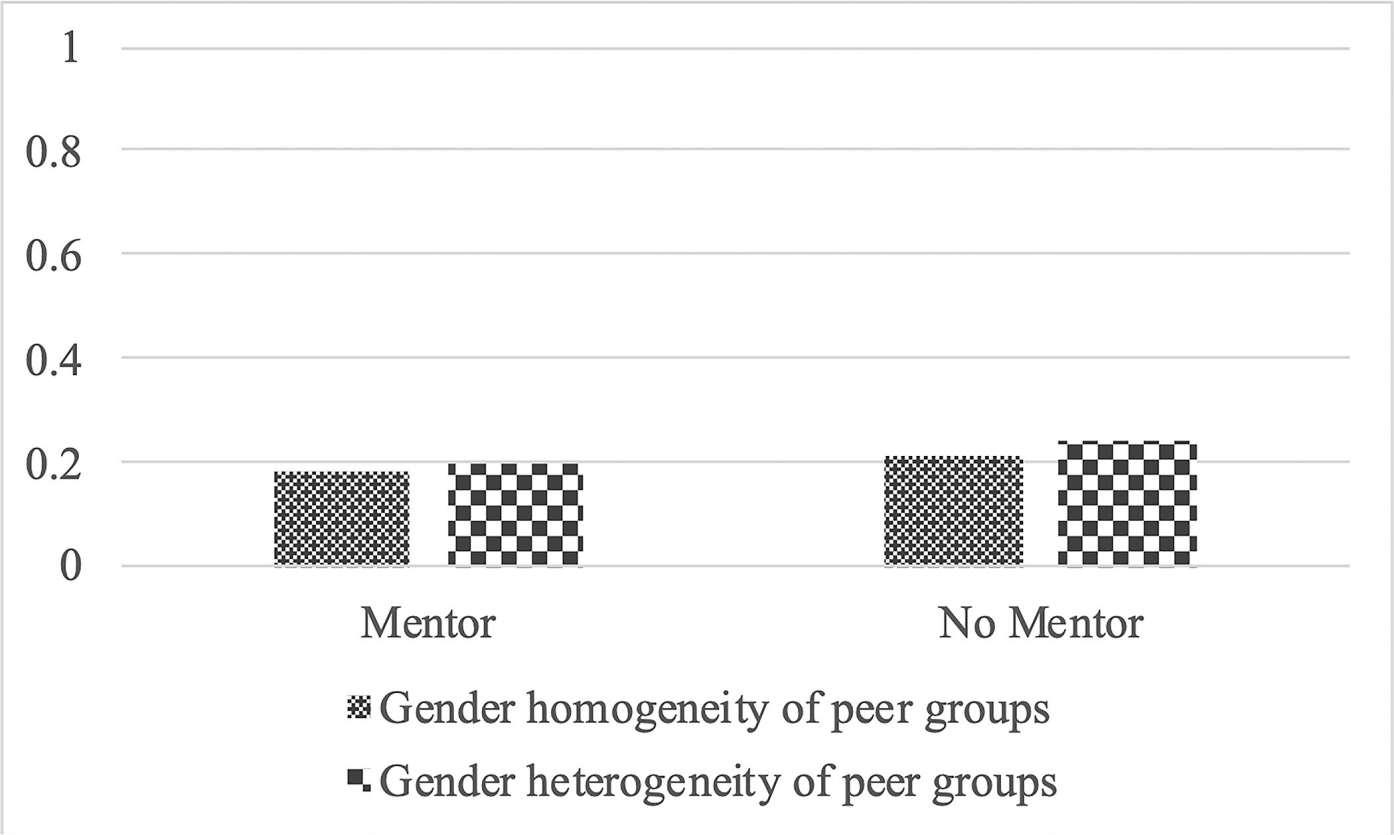
Predictive margins of suicidal ideation by mentor availability and the gender heterogeneity of peer groups among boys.

**Fig 3:**
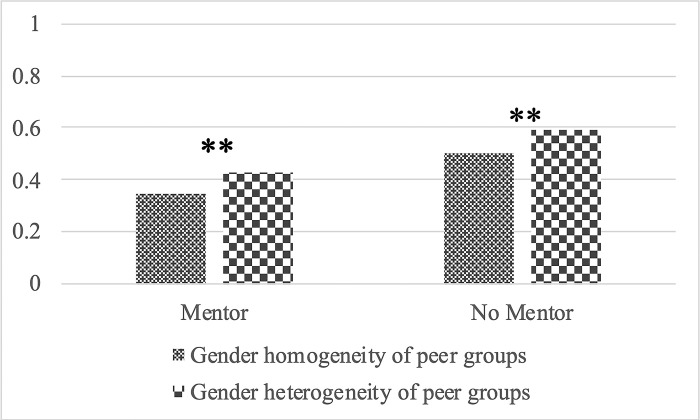
Predictive margins of suicidal ideation by mentor availability and the gender heterogeneity of peer groups among girls. ** p < 0.01.

Fig 2 for boys and Fig 3 for girls illustrate how the gender heterogeneity of peer groups and having a mentor intersect with the probability of suicidal ideation. Fig 2 shows that among boys with a mentor, there is no significant difference in the probability of suicidal ideation regardless of the gender heterogeneity of peer groups. Similarly, the gender heterogeneity of peer groups does not produce a statistically significant difference among boys without a mentor. In contrast, Fig 3 indicates that among girls with a mentor, those with male and female friends have a higher probability of suicidality than those with female friends only. However, among girls without a mentor, the likelihood of suicidal ideation is not significantly different by the gender heterogeneity of peer groups. These findings suggest support for *Hypothesis 2*.

## Discussion

Despite voluminous studies on risk factors for adolescent suicidality [56–59], little research examines the role of peer characteristics in suicidal ideation. During adolescence, the role of friends is influential, and many researchers have investigated how it is related to physical and mental health outcomes [60, 61]. Several studies have revealed the importance of friend relationships in suicidal ideation [51, 56], but the gender heterogeneity of peer groups has rarely been examined [62]. The present study demonstrated that the gender heterogeneity of friends is associated with suicidal ideation in boys and girls. This study found that adolescents with friends of different genders tend to have a higher possibility of suicidal ideation. This finding implies that different-gender friends are less effective in alleviating their friends’ concerns. More importantly, the association is more apparent among girls. Additionally, having a mentor significantly reduces the likelihood of suicidality when girls have only female friends compared to when girls have both male and female friends. These results suggest that girls may be more actively supportive and responsive when their peer groups consist of girls only. This finding is consistent with previous studies that reveal supportive attitudes of girls [31, 33, 34] and greater effectiveness in female cliques [37–39]. Girls might receive much stronger collective support from a cohesive group of female-only friends. The advantages of gender homogeneity are also found in education through improved academic achievement [63].

A remaining question involves the role of male friends in suicidal ideation for girls. One study reported that boys in mixed-gender friendships influenced the behavior of their female friends, while girls did not show such an influence [64]. As described in the introduction, boys may avoid emotional issues by disregarding or ridiculing them [41]. Under these circumstances, girls may be less expressive in social support than they are in female groups. Another study noted a greater risk of poor academic outcomes among girls when they were exposed to mixed-gender peers at the class level [65].

Regarding the initial question of this study, these findings provide a potential explanation for why Korean girls’ suicide rate is unexpectedly high compared to boys in Korea and even compared to girls in advanced countries. Girls with male and female friends may not benefit from increased social solidarity, which may be notable among all-girl peer groups. In Korea, the gender composition of adolescents’ peer groups may have changed. Although there are no direct data showing the trend change in the gender composition of peer groups, one potential clue is the continuous increase in the number of coeducational schools. In 1999, 60.2% of middle schools and 40.1% of high schools were coeducational schools [66]. However, even in coeducational schools, boys and girls tend to be separated by assigning them to different classrooms, although this trend has weakened since the late 1990s. As of 2022, the percentages of coeducational schools had increased by approximately 20% for middle and high schools [66]. In parallel with this change, gender segregation in friendship networks among Korean adolescents might also become a less explicit norm. The decline in gender segregation should be accepted as an achievement because segregation can intensify gender stereotypes and bias [67]. However, girls may experience an unexpected decrease in social support through interventions for gender integration. Therefore, further examination of whether a change in the gender composition of peer groups affects the levels of social support, suicidal ideation, and suicidal attempts is needed.

There may be a detrimental effect for girls of having male friends, and this effect might be directly associated with the relatively higher suicide rate of Korean girls. Male friends might facilitate the execution of suicide because boys have indicated a higher suicide mortality rate, partially due to the use of more lethal methods [68]. The significance of peers in determining adolescent risk behaviors has been widely confirmed [69–71]. However, further investigation is necessary to apply this explanation to suicide among Korean girls.

The current study has several limitations. Friend networks may also transmit suicidal ideation [35, 49, 72]. However, this study could not test whether suicidality diffuses among peer groups consisting of girls due to the inaccessibility of such information. This type of data would help examine how peer groups deal with suicidal ideation as a topic of conversation. Another limitation is that the current dataset did not specify the details of different-gender friends. Respondents provided only their best friends’ names/nicknames, gender, social class, and academic grade. Friends of different genders could be ’friends’ or ’boyfriends or girlfriends.’ This difference may affect the association between different-gender friends and suicidal ideation. For instance, one study reported that adolescents with romantic relationships tend to have a greater likelihood of juvenile delinquency [73]. The data also do not provide information on the degree of masculinity or femininity of girls’ male friends. We assumed that these boys would maintain their masculinity in relationships with girls; however, their gender role orientation might already be close to femininity due to their connection to girls [74]. In this sense, these boys are likely to be more supportive of their female friends than boys with only male friends. This study also considered a wide range of covariates that were suggested by prior research, but two critical variables were still lacking. One variable is depression, which is the most significant factor for suicidal ideation among adolescents [8–12]. To address this, we included loneliness and stress variables as alternatives, but adjusting for depression might influence the results. The other variable is menarche. A recent study suggested that early menarche is associated with suicidality [20] and is specific to girls. Therefore, controlling for this variable may affect the relationship between the gender heterogeneity of peer groups and suicidal ideation among girls. Finally, we suggested that the change in gender composition of peer groups among girls due to the increase in coeducational schools might decrease the level of social support that girls receive, which might be associated with a greater level of suicidality. However, the present study was based on cross-sectional data, not longitudinal data. Future studies that examine multiple time points of respondents could better address these pathways.

Despite these limitations, this study contributes significantly to the literature on adolescent suicidal ideation and peer effects. While girls in Korea tend to be more stressed and less satisfied with their relationships than boys, their gender heterogeneity of friends is likely to increase the chance of suicidal ideation. School counselors and practitioners should be alert to girls’ peer characteristics during adolescent suicidality consultations.

## Supporting information

S1 Checklist STROBE statement—checklist of items that should be included in reports of observational studies. (PDF)

S1 Fig ROC curve analysis. (TIFF)

S1 Table Specification error. (PDF)

S2 Table Multicollinearity check. (PDF)

S3 Table Missingness. (PDF)
