# Supplementary material for: Why is Korean girls’ suicidal ideation rate higher than boys’ rate? The role of gender heterogeneity in peer groups
Source: PLoS One. 2023 Sep 6;18(9):e0290072. doi: 10.1371/journal.pone.0290072 (PMC10482302; doi:10.1371/journal.pone.0290072)
Supplement: S1 Checklist — (PDF) [file pone.0290072.s001.pdf]

STROBE Statement—checklist of items that should be included in reports of observational studies

	Item No.	Recommendation	Page No.	Relevant text from manuscript
**Title and abstract**	1	(*a*) Indicate the study's design with a commonly used term in the title or the abstract	p.2	For analysis, logistic regression models with survey weights were used.
		(*b*) Provide in the abstract an informative and balanced summary of what was done and what was found	p.2	Adjusting for the covariate, the analysis revealed that adolescents with different-gender friends were associated with a higher likelihood of suicidal ideation than those with exclusively same-gender friends. In addition, an analysis stratified by gender found this association only significant among girls. Furthermore, the protective power of a mentor against suicidal ideation was significantly lower in girls with male and female friends than in girls with only female friends.
**Introduction**				
Background/rationale	2	Explain the scientific background and rationale for the investigation being reported	p.3 – p.4	… weakened social support due to the gender heterogeneity of peer groups among girls may contribute to an increased likelihood of suicidal ideation…
Objectives	3	State specific objectives, including any prespecified hypotheses	p.4	… the present study investigated an association

				between the gender heterogeneity of the peer groups and suicidal ideation. On the basis of the literature, we constructed the following hypotheses…
**Methods**				
Study design	4	Present key elements of study design early in the paper	p.4	… the present study investigated an association between the gender heterogeneity of the peer groups and suicidal ideation…
Setting	5	Describe the setting, locations, and relevant dates, including periods of recruitment, exposure, follow-up, and data collection	p.5	The survey collected data from middle and high school students between March 7th, 2018 and April 6th, 2018.
Participants	6	(*a*) *Cohort study*—Give the eligibility criteria, and the sources and methods of selection of participants. Describe methods of follow-up<br>*Case-control study*—Give the eligibility criteria, and the sources and methods of case ascertainment and control selection. Give the rationale for the choice of cases and controls<br>*Cross-sectional study*—Give the eligibility criteria, and the sources and methods of selection of participants	p.5	Participants were chosen by Probability Proportional to Size (PPS) sampling regarding school, region, and sex while considering a population unit as a class.
		(*b*) *Cohort study*—For matched studies, give matching criteria and number of exposed and unexposed<br>*Case-control study*—For matched studies, give matching criteria and the number of controls per case	n/a	
Variables	7	Clearly define all outcomes, exposures, predictors, potential confounders, and effect modifiers. Give diagnostic criteria, if applicable	p.5 – p.8	e.g., The outcome variable is whether a person had an impulse to commit suicide…
Data sources/ measurement	8*	For each variable of interest, give sources of data and details of methods of assessment (measurement). Describe comparability of assessment methods if there is more than one group	p.5 – p.8	e.g., The survey asked if a participant had ever thought

				about their suicide by impulse (yes = 1; no = 0) …
Bias	9	Describe any efforts to address potential sources of bias	p.5	Among the questions, approximately 20% of respondents did not answer their best friends’ levels of academic grades. However, a sensitivity test excluding this item still produced similar results in full models adjusting for confounders.
Study size	10	Explain how the study size was arrived at	p.5	After removing those with missing values, the analytic sample includes 2,990 students in middle and high schools, consisting of 1,602 boys and 1,388 girls.

Continued on next page

Quantitative variables	11	Explain how quantitative variables were handled in the analyses. If applicable, describe which groupings were chosen and why	p.5 – p.8	e.g., Respondents were asked if they felt loneliness and answered it on a 5-point scale. Greater scores demonstrate lonelier status.
Statistical methods	12	(*a*) Describe all statistical methods, including those used to control for confounding	p.8	A logistic regression framework with survey weights was adopted for the binary dependent variable of whether a person had an impulse to commit suicide…
		(*b*) Describe any methods used to examine subgroups and interactions	p.8	Interaction terms in logistic regression models need caution in interpreting the size and direction of estimates and statistical significance. Instead, a delta method for statistical significance can be used more appropriately.
		(*c*) Explain how missing data were addressed	p.5	After removing those with missing values, the analytic sample includes 2,990 students in middle and high schools, consisting of 1,602 boys and 1,388 girls.
		(*d*) *Cohort study*—If applicable, explain how loss to follow-up was addressed<br>*Case-control study*—If applicable, explain how matching of cases and controls was addressed<br>*Cross-sectional study*—If applicable, describe analytical methods taking account of sampling strategy	p.8	A logistic regression framework with survey weights was adopted for the binary dependent variable of whether a person had an impulse to commit suicide.
		(*e*) Describe any sensitivity analyses	p.9	Diagnostic tests were employed and reported as supporting information.
**Results**				
Participants	13*	(a) Report numbers of individuals at each stage of study—eg numbers potentially eligible, examined for eligibility, confirmed eligible, included in the study, completing follow-up, and analysed	p.9	Please see Table 1.

		(b) Give reasons for non-participation at each stage		We used a maximum number of participants after considering potential confounders.
		(c) Consider use of a flow diagram		Because our selection process of participants was simple, a flow diagram was not used.
Descriptive data	14*	(a) Give characteristics of study participants (eg demographic, clinical, social) and information on exposures and potential confounders	p.9	Please see Table 1.
		(b) Indicate number of participants with missing data for each variable of interest		Please see Supporting Information.
		(c) *Cohort study*—Summarise follow-up time (eg, average and total amount)	n/a	
Outcome data	15*	*Cohort study*—Report numbers of outcome events or summary measures over time	n/a	
		Case-control study—Report numbers in each exposure category, or summary measures of exposure	n/a	
		Cross-sectional study—Report numbers of outcome events or summary measures	p.9	Please see Table 1.
Main results	16	(*a*) Give unadjusted estimates and, if applicable, confounder-adjusted estimates and their precision (eg, 95% confidence interval). Make clear which confounders were adjusted for and why they were included	p.13 & p.16	Please see Table 2 and 3.
		(*b*) Report category boundaries when continuous variables were categorized		Continuous variables were not used as categorical.
		(*c*) If relevant, consider translating estimates of relative risk into absolute risk for a meaningful time period		Because the analysis estimated a likelihood of suicidal ideation, relative risk was reported.

Continued on next page

Other analyses	17	Report other analyses done—eg analyses of subgroups and interactions, and sensitivity analyses	p.9	No specification errors or multicollinearity were found. Also, please refer to Supporting Information.
**Discussion**				
Key results	18	Summarise key results with reference to study objectives	p.18	This study found that adolescents with friends of different gender tend to have a higher possibility of suicidal ideation…
Limitations	19	Discuss limitations of the study, taking into account sources of potential bias or imprecision. Discuss both direction and magnitude of any potential bias	p.19	Friend networks may also transmit suicidal ideation… Another limitation is that the current dataset does not specify the details of different-gender friends…
Interpretation	20	Give a cautious overall interpretation of results considering objectives, limitations, multiplicity of analyses, results from similar studies, and other relevant evidence	p.18	The advantages of gender homogeneity are also found in education through improved academic achievement.
Generalisability	21	Discuss the generalisability (external validity) of the study results	p.18	… girls may experience an unexpected decrease in social support through intervention for gender integration.
**Other information**				
Funding	22	Give the source of funding and the role of the funders for the present study and, if applicable, for the original study on which the present article is based	n/a	

*Give information separately for cases and controls in case-control studies and, if applicable, for exposed and unexposed groups in cohort and cross-sectional studies.

Note: An Explanation and Elaboration article discusses each checklist item and gives methodological background and published examples of transparent reporting. The STROBE checklist is best used in conjunction with this article (freely available on the Web sites of PLoS Medicine at http://www.plosmedicine.org/, Annals of Internal Medicine at http://www.annals.org/, and Epidemiology at http://www.epidem.com/). Information on the STROBE Initiative is available at www.strobe-statement.org.
