# Supplementary figures and images for: Why is Korean girls’ suicidal ideation rate higher than boys’ rate? The role of gender heterogeneity in peer groups
Source: PLoS One. 2023 Sep 6;18(9):e0290072. doi: 10.1371/journal.pone.0290072 (PMC10482302; doi:10.1371/journal.pone.0290072)

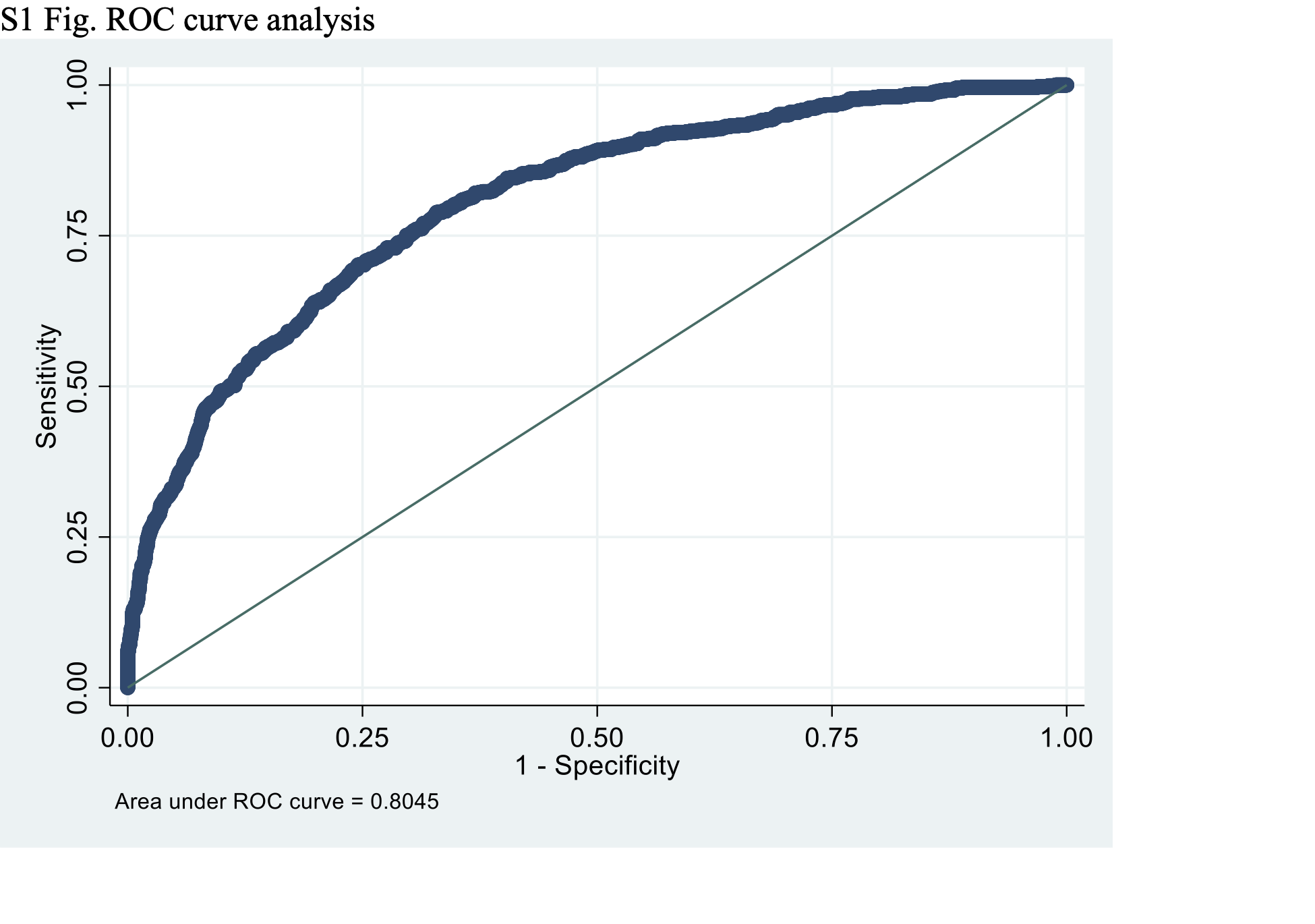

Supplement: S1 Fig — (TIFF) [file pone.0290072.s002.tiff]
