# Supplementary material for: Why is Korean girls’ suicidal ideation rate higher than boys’ rate? The role of gender heterogeneity in peer groups
Source: PLoS One. 2023 Sep 6;18(9):e0290072. doi: 10.1371/journal.pone.0290072 (PMC10482302; doi:10.1371/journal.pone.0290072)
Supplement: S1 Table — (PDF) [file pone.0290072.s003.pdf]

S1 Table. Specification error

Suicidal ideation	Coef.	Std. Err.	z	P > |z|	[95% Conf. Interval]	
Hat	1.03	0.06	16.41	0.00	0.91	1.16
Hat square	0.02	0.03	0.77	0.44	-0.03	0.08
cons	-0.01	0.06	-0.20	0.85	-0.13	0.11
