# Supplementary material for: Why is Korean girls’ suicidal ideation rate higher than boys’ rate? The role of gender heterogeneity in peer groups
Source: PLoS One. 2023 Sep 6;18(9):e0290072. doi: 10.1371/journal.pone.0290072 (PMC10482302; doi:10.1371/journal.pone.0290072)
Supplement: S2 Table — (PDF) [file pone.0290072.s004.pdf]

S2 Table. Multicollinearity check

Variable	VIF	SqRT VIF	Tolerance	R-Squared
Girls	1.44	1.20	0.69	0.31
# of best friends	1.19	1.09	0.84	0.16
Same gender	1.10	1.05	0.91	0.10
Same social class	1.07	1.04	0.93	0.07
Same grade	1.09	1.04	0.92	0.08
Mentor	1.12	1.06	0.90	0.10
Friends: I feel ashamed	1.41	1.19	0.71	0.29
Friends: I feel lonely	1.74	1.32	0.58	0.43
Friends: I feel anger	1.53	1.24	0.65	0.35
Friends: No care	1.63	1.28	0.61	0.39
Bullied	1.16	1.08	0.86	0.14
Bullying	1.13	1.06	0.88	0.12
Parents: Conflict	2.81	1.68	0.36	0.64
Parents: Interference	2.46	1.57	0.41	0.59
Parents: Lack of understanding	2.82	1.68	0.35	0.65
Good relationship with a father	1.45	1.20	0.69	0.31
Good relationship with a mother	1.48	1.22	0.67	0.33
Good relationship between parents	1.33	1.16	0.75	0.25
Loneliness	1.42	1.19	0.71	0.29
Drinking	1.24	1.11	0.81	0.19
Smoking	1.24	1.11	0.81	0.19
Sexual intercourse	1.17	1.08	0.86	0.14
Stress: Bad grades	2.01	1.42	0.50	0.50
Stress: Exams	1.82	1.35	0.55	0.45
Gaming	1.21	1.10	0.82	0.18
Workout	1.07	1.03	0.94	0.06
Club activities	1.24	1.11	0.81	0.19
Participation in religious service	1.13	1.06	0.88	0.12
Volunteering	1.35	1.16	0.74	0.26
Participation in civil activities	1.38	1.18	0.72	0.28
Celebs fan club	1.25	1.12	0.80	0.20
Middle schooler	1.37	1.17	0.73	0.27
Social class	1.10	1.05	0.91	0.09
Self-reported health	1.34	1.16	0.75	0.25
Pharmacy visit	1.07	1.03	0.94	0.06
Hospitalization	1.05	1.02	0.95	0.05
Sleep time	1.07	1.03	0.94	0.06
Mean VIF	1.40			
