# Supplementary material for: Why is Korean girls’ suicidal ideation rate higher than boys’ rate? The role of gender heterogeneity in peer groups
Source: PLoS One. 2023 Sep 6;18(9):e0290072. doi: 10.1371/journal.pone.0290072 (PMC10482302; doi:10.1371/journal.pone.0290072)
Supplement: S3 Table — (PDF) [file pone.0290072.s005.pdf]

S3 Table. Missingness

	Missing	Percent missing
Suicidal ideation (%)	77	1.5
Gender	0	0
Number of best friends (0-7)	0	0
Gender heterogeneity of peer groups (%)	229	4.5
Academic achievement heterogeneity of peer groups (%)	1,052	20.5
Social class heterogeneity of peer groups (%)	262	5.1
I have someone to discuss my concerns (%).	119	2.3
Relationship with best friends (all: 1-5 scale)		
I feel ashamed to talk about my problems to them.	200	3.9
I feel lonely even while having time with them.	202	3.9
I feel anger due to them.	202	3.9
They do not care about my sorrow.	203	4.0
A good relationship with a father (1-5)	296	5.8
A good relationship with a mother (1-5)	222	4.3
A good relationship between father and mother (1-5)	471	9.2
Loneliness (1-5)	11	0.2
Bullied (%)	14	0.3
Bullying (%)	7	0.1
Drinking ever (%)	10	0.2
Smoking within a week (%)	6	0.1
Sexual intercourse ever (%)	187	3.6
Stress from conflicts with parents (1-5)	108	2.1
Stress from parental interference (1-5)	107	2.1
Stress from lack of understanding by parents (1-5)	105	2.0
Stress from bad grades (1-5)	84	1.6
Stress from assignments or exams in school (1-5)	82	1.6
Club activities (1-5)	15	0.3
Participation in religious services (1-5)	17	0.3
Volunteering activities (1-5)	14	0.3
Participation in civic activities (1-5)	15	0.3
Celebs fan club (1-5)	16	0.3
Gaming (1-5)	409	8.0
Workout days a week (0-7)	50	1.0
Sleep time (Centered on group means: middle and high school, each)	71	1.4

Self-reported health (1-5)	7	0.1
Visit to a pharmacy within two weeks (%)	4	0.1
Hospitalization in a year (%)	5	0.1
Middle schoolers: ref. high schoolers (%)	0	0
Academic achievement (1-3)	0	0
Social class (1-3)	80	1.6
